# Stress relaxation in the presence of sudden strain bursts: Methodology and stress relaxation data of microcast aluminium microwires

**DOI:** 10.1016/j.dib.2018.11.047

**Published:** 2018-11-14

**Authors:** S. Verheyden, L. Deillon, A. Mortensen

**Affiliations:** Ecole Polytechnique Fédérale de Lausanne (EPFL), Switzerland

## Abstract

This data article presents a methodology and the corresponding code developed to perform and process stress relaxation tests where samples display superimposed (i) classical, continuous logarithmic relaxation together with (ii) sudden displacements manifest as abrupt stress decreases. The method extracts the activation area characteristic of the thermally activated mechanism that drives continuous plastic deformation in the material. We report stress relaxation data appertaining to as-cast (27) and annealed (2) aluminium microwires produced through a microcasting process. For an interpretation and discussion of the data on annealed microwires the reader is referred to “ The effect of size on the plastic deformation of annealed cast aluminium microwires” (Verheyden et al., In Press) [1]. For full descriptions of the production process of aluminium microwires or of the tensile testing equipment and procedure the reader is referred to Krebs et al. (2017) [2].

## Specifications table

**Table:** 

Subject area	*Materials science, metallurgy*
More specific subject area	*Size effect, thermal activation*
Type of data	*Table, figure, graphs*
How data was acquired	*Lab-built microtensile machine with 10 g or 50 g LPM200 Futek loadcell (Irvine, California)*
Data format	*Filtered and analysed*
Experimental factors	*As-cast: no pre-treatment*
	*Annealed: microwires are encapsulated and annealed for 2 h at 500 °C under a protective Argon atmosphere.*
Experimental features	*Individual stress relaxation tests allow for the extraction of the apparent activation area, a fingerprint of the mechanism controlling thermally activated deformation in a material.*
Data source location	*Laboratory of Mechanical Metallurgy at EPFL*
Data accessibility	*Data is included in this article*
Related research article	S. Verheyden, L. Pires, L. Deillon, A. Mortensen, The effect of size on the plastic deformation of annealed cast aluminium microwires, Scr. Mater. (In Press) [1]

## Value of the data

• The data present stress relaxations containing sudden displacement events and can be used to study the plasticity size effect.
• The described method can be used to determine the apparent activation area from the continuous logarithmic part of a stress relaxation curve when such a curve displays jumps caused by sudden displacement events
• Raw test data, as well as treated test data giving activation area values, can be used for comparison with other studies on the role of thermal activation in the plastic deformation of small (< 100 µm) crystals.

## 1 Data

This dataset includes the datafiles (.txt) of the individual stress relaxations performed on aluminium microwires (99.99% and 99.999% purity) in one of two states: (i) as-cast state or (ii) annealed (sample description in Material section). The general set-up and experimental parameters are described in Methods. The plastic deformation of monocrystals with a dimension < 100 µm is intermittent, also during stress relaxation. Therefore a methodology is introduced that separates the continuous part of the stress relaxation signal from its intermittent part. This methodology includes a list of criteria to keep or discard stress relaxations as described below. For each tested wire a folder containing all the stress relaxations is provided as well as a Mathematica^®^ notebook that performs the described calculations. The extracted values as well as the final retained datasets can be found in the ‘final results’ folder.

## 2 Experimental design, materials and methods

### 2.1 Materials: list of samples

See Tables 1 and 2.

**Table 1:** Overview of the characteristics of as-cast aluminium microwires. Includes the number of individual stress relaxations performed.

**Sample name**	**Material**	**# of relaxations**	***D*****[µm]**	**Orientation**	**L**_**0**_**[µm]**
**Al_20_r_9**	Al4N	8	14.3	[-2 7 1]	1674
**Al_20_r_10**	Al4N	8	14.7	[7 -1 -2]	1572
**Al_20_r_11**	Al4N	9	14.3	[8 -1 -2]	1843
**Al_20_r_14**	Al4N	18	14.3	[4 5 2]	818
**Al_20_r_15**	Al4N	9	13.6	[2 3 -6]	1359
**Al_20_r_16**	Al4N	21	14.4	[4 5 -2]	1104
**Al_20_r_18**	Al4N	8	12.7	[-3 6 -1]	1384
**Al_20_r_19**	Al4N	11	14.6	[0 -4 -3]	1409
**Al_20_r_25**	Al4N	37	14.7	[1 1 1]	1393
**Al_20_r_28**	Al4N	13	14.5	[2 2 -3]	1325
**Al_27_r_7**	Al4N	37	24.2	[-3 3 4]	1455
**Al_27_r_8**	Al4N	59	25.0	[3 3 4]	1140
**Al_27_r_9**	Al4N	18	22.2	[3 1 7]	1104
**Al_27_r_10**	Al4N	15	22.5	[2 5 0]	1463
**Al_27_r_12**	Al4N	19	24.0	[-1 5 1]	1111
**Al_27_r_13**	Al4N	37	23.1	[1 5 -5]	1281
**Al_100_r_1**	Al4N	48	121	[4 2 5]	1440
**Al_100_r_3**	Al4N	29	115	[4 8 -1]	987
**5N_27_r_1**	Al5N	43	24.0	[2 2 1]	1473
**5N_27_r_2**	Al5N	23	24.3	[2 -3 -1]	1768
**5N_27_r_3**	Al5N	19	24.0	[6 2 -3]	787
**5N_27_r_4**	Al5N	20	21.6	[2 4 -5]	1964
**5N_100_r_4**	Al5N	38	114	[4 1 -6]	1442
**5N_100_r_7**	Al5N	22	125	[-3 0 -5]	1859
**5N_100_r_8**	Al5N	35	114	[-3 5 0]	2280
**5N_100_r_9**	Al5N	24	116	[1 1 0]	1657
**5N_100_r_10**	Al5N	24	116	[8 3 -1]	2026

**Table 2:** Overview of the characteristics of annealed aluminium microwires. Includes the number of individual stress relaxations performed.

**Sample name**	**Material**	**# of relaxations**	***D*****[µm]**	**Orientation**	***L***_**0**_**[µm]**
**AN_20_3**	Al4N	11	14.0	[5 3 1]	1545
**AN_27_2**	Al4N	23	23.7	[7 -2 -3]	1517

### 2.2 Experimental design, materials and methods

#### 2.2.1 Setup

**Fig. 1:**
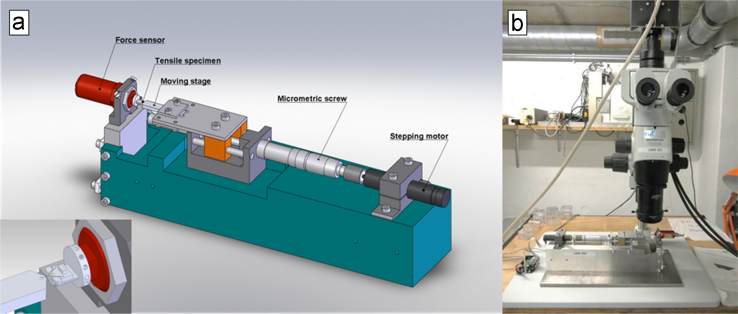
a) Illustration of tensile bench with inset showing the gauge area. b) The tensile bench as mounted beneath a binocular and camera [3].

All tensile tests are performed using the custom-built displacement-controlled tensile machine described in Ref. [2], [3] and shown in Fig. 1. The gauge area of the tensile bench is located under a binocular (Olympus SZX16) equipped with a camera. The imposed displacement cycle as well as the data acquisition are controlled by means of a LabVIEW^®^ code.

Three different datasets are acquired during a typical test: time–displacement–force data acquired at 50 Hz, time-force data acquired at 50 kHz and optical images of the gauge length, acquired at 2 Hz. All apparent activation area calculations are based on the 50 Hz data.

A 10 g Futek (Irvine, USA) LPM 200 load cell is used for microwires with *D* < 50 µm while a 50 g Futek LPM200 load cell is used when *D* > 50 µm. For all tests a displacement rate of 300 nm/s is imposed. A more detailed description of the machine and its parameters can be found in Krebs et al. [3]. The 50 kHz time-force data are filtered four times by means of a moving average filter over 250 points (approximating a Gaussian filter [4], made somewhat stricter here than in Ref. [3] where 160 points were used). The stability of the set-up was initially checked using stiffer dummy samples, from which the tensile bench was deemed sufficiently stiff and free of time-dependent deformation mechanisms for relaxation testing to generate meaningful data.

During a stress relaxation test, the sample is loaded up to a pre-defined stress or strain level at which point the cross-head of the machine is stopped; the (decreasing) stress is then recorded versus time [5], [6]. Within this work, individual 60 s long stress relaxations were triggered at predefined load levels. The number of stress relaxations performed on a given microwire therefore depends (among other parameters) on the amount of work hardening during the test.

#### 2.2.2 Method: extracting the apparent activation area

This method focuses on the continuous parts of the stress relaxations and calculates the apparent activation area based on this. The method implements a list of mathematical criteria that identify and omit the intermittent parts from the stress relaxations.

The apparent activation area of a stress relaxation can be calculated using Eq. (1).

(1) $$a=\frac{\mathrm{kT}}{b}\frac{\partial \ln(-\dot{\tau }/M)}{\partial \tau }$$

With *M* the stiffness of the machine-wire system [MPa/s].

The binning method consists of 3 main steps: (1) binning the stress relaxation data to smoothen the time derivative, (2) discard bins that correspond to sudden displacement jumps and (3) calculate the apparent activation area based on the remaining bins. For each microwire these three steps are performed in the Mathematica^®^ notebook (samplename_doublederivativemethod.nb) and its packages that can be found in the folder containing the stress relaxation data. The notebook can be used to extract the activation area from all stress relaxations of a single microwire at once.

#### 2.2.3 Binning of a relaxation

**Fig. 2:**
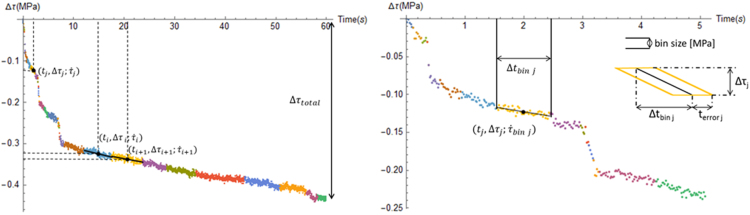
(left) Example of a binned (60 s) relaxation including a linear fit through three different bins. (right) zoom-in on the first 5 s of the relaxation, with a focus on the parameters used to characterize a bin.

The change in stress as a function of time is registered at a 50 Hz frequency. Since individual relaxations are 60 s long, each stress relaxation produces roughly 3000 data points. To calculate the time derivative ($\dot{\tau }$) of a relaxation curve, the data are first binned into segments sufficiently small to be approximated as straight lines. The logarithmic nature of a continuous relaxation curve implies that there will be an initial rapid decrease in stress with time and a near-horizontal signal towards longer relaxation times. This means that the bin size (in terms of number of data points) needs to change accordingly to capture this change in slope (smaller initial bins, larger bins at the end). To this end, the data are binned in stress-defined equi-sized bins. The bin size is taken to be either 1.5*σ_τ_* or Δ*τ_total_*/30, whichever is larger. The first criterion considers that the bin size needs to be larger than the standard deviation (measure of noise) on the stress signal (*σ_τ_*). The second criterion limits the total number of bins to 30. In order to define a linear fit within each bin, the points need to be sequential in time. Therefore the size of the consecutive stress-defined bins (i.e., the number of data points in each bin) is used to define the final partitioning of the relaxation. An example of a binned dataset is given in the left-hand figure below (Fig. 2).

#### 2.2.4 Selection of bins appertaining to the continuous decrease in stress

To calculate the activation area of the continuous part of the relaxation, data pertaining to jumps (and the bins they are in) should not be included. To discard all bins containing a list of criteria was defined; the process is fully automated in the joined Mathematica^®^ notebooks. A bin is discarded if:

1. it is too short for a linear regression to be defined with confidence, namely, if it contains less than 5 data points.
2. if $\dot{\tau }>0$ and thus $\ln(-\dot{\tau }/M)$ is not defined.
3. if $\overset{˙}{\tau }<-1\;\mathrm{MPa}/s$ (this high rate of stress decrease being an absolute and clear criterion for the presence of a jump).
4. if $\frac{\sigma_{\tau }/∆t_{\mathrm{bin}}}{\dot{\tau }}<-1$ (if the measured slope is less than the standard deviation of the noise (*σ_τ_*) in the data divided by the bin length, it has little meaning).
5. if $\frac{\sigma_{\tau }}{∆t_{\mathrm{bin i}+1}+t_{\mathrm{error i}+1}}<\frac{\sigma_{\tau }}{∆t_{\mathrm{bin i}}+t_{\mathrm{error i}}}$ with $t_{\mathrm{error}}=\frac{\sigma_{\tau }\Delta t_{\mathrm{bin}}}{\Delta \tau_{\mathrm{bin}}}$ (the slope should, within uncertainty caused by noise, decrease with stress or in other words bins should become larger with relaxation time).
6. if $\frac{∆\tau_{\mathrm{bin i}+1}-∆\tau_{\mathrm{bin i}}}{∆\tau_{\mathrm{total}}}>0.5$ (following very large jumps the relaxation may show negative stress relaxation similar to what is observed during stress reduction experiments [5], [7]. To avoid any effect of this the two subsequent bins are discarded).

**Fig. 3:**
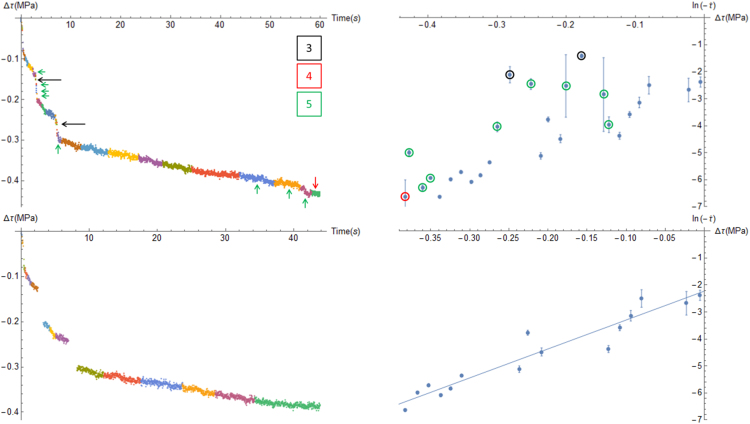
(Top) A binned relaxation from which, based on numbered selection criteria listed in the main text, bins that do not appertain to the continuous part of the relaxation are discarded and corresponding $∆\tau -\ln(-\overset{˙}{\tau })$ plot. (Bottom) the remaining bins representing the thus identified continuous part of the relaxation and the linear fit through the $∆\tau -\ln(-\overset{˙}{\tau })$ plot. Note that the bins are plotted in a reversed order on the $∆\tau -\ln(-\overset{˙}{\tau )}$ plot.

The effect of the three last of these criteria on the bins used, and the corresponding remaining points in the dataset, is illustrated in Fig. 3. The different criteria are colour-coded to illustrate which bin (marked with a coloured arrow) is discarded for which reason. The process is fully automated in the joined Mathematica^®^ notebooks.

#### 2.2.5 Selection of a relaxation

Although it is mathematically possible to calculate an activation area using the present (binning) method for every stress relaxation, this value is not always trustworthy, as the code will extract a value regardless of the extent or plausibility of data points. The following relaxations are deemed unfit to yield a valid activation area value:

• Calculation based on less than 3 bins.
• The stress range covered by the remaining bins represents less than 20% of the overall stress decrease during the relaxation.
• The calculated error is larger than the actual value.

The final removal of stress relaxations inadequate to extract an apparent activation area is done in the joined excel file for each microwire.

#### 2.2.6 Calculating the activation area and the error on the activation area

During the derivation of the activation area from the time-stress data 2 linear regressions are performed, namely one to extract the time-derivative of the stress within each bin of a given relaxation and one to extract the apparent activation area from the logarithm of the derivative of τ for each relaxation. The activation area is inverted for plotting in the Haasen plot. Initial error on the signal will give rise to an error on the first regression (when calculating $\dot{\tau }$). This error is taken into account in further calculation steps when calculating error in the final data.

Additionally, because relaxation data are measured using a load cell, they will inherently suffer from white noise (Gaussian, the same for all measured *τ_i_* values). $\dot{\tau }$ and $\sigma_{\dot{\tau }}$ are calculated for each of the bins using a linear regression through the points in the relevant bin. The stress value (*τ*) at the calculated point is taken to be the average stress over the bin. The standard deviation of the estimated value of $\dot{\tau }$ is given by Eq. (2). The implicit assumption is made that the measured error is normally distributed (i.e., that it is white noise) and the same for every stress measurement [8].

(2) $$\sigma_{\dot{\tau }}=\sqrt{\frac{1}{(N-2)}∙\frac{\sum_{i=1}^{N}{\left(\tau_{i}-\tau_{\mathrm{fit}}\right)}^{2}}{\sum_{i=1}^{N}(t_{i}-{\bar{t})}^{2}}}$$

The calculated values then need to be rewritten as $\ln\;(-\dot{\tau })$. This will transform the error as follows:

(3) $$\sigma_{\ln\;(-\dot{\tau })}=\left(-1\right)\;∙\;\sigma_{\dot{\tau }}$$

(The negative sign accounts for the fact that an upper error becomes a lower error and vice versa) [8].

We now have a set of $((\tau_{i},\ln\left(-{\dot{\tau }}_{i}\right));$ $\sigma_{\ln\;(-\dot{\tau })}$) data points from which we want to extract, by linear regression, the slope in the $\ln\left(-\dot{\tau }/M\right)$ versus Δ*τ* plot to extract the activation area (*a*). This is a linear plot if continuous relaxation kinetics obey the often logarithmic stress decay law. Since we know the error on individual points on that plot, we can estimate their effect on the slope. In the present instance, one must take into account, in the linear regression, the fact that the error associated with each point is not the same (for the exact equations see p. 105 Eq. (6.12) of Ref. [8]).

The error on the calculated slope will, thus, depend on the (variable) error on the fitted points:

(4) $$\sigma_{\mathrm{slope}}=\sqrt{\frac{1}{\Delta }\sum_{i=1}^{N}\frac{1}{\sigma_{i}^{2}}}$$

(5) $$\Delta =\sum \frac{1}{\sigma_{i}^{2}}\sum \frac{x_{i}^{2}}{\sigma_{i}^{2}}-{(\sum \frac{x_{i}}{\sigma_{i}^{2}})}^{2}$$

The slope calculated in this manner is directly related to *Δa* as:

(6) $$a=\frac{\mathrm{kT}}{b}∙\frac{\partial \ln\left(-\frac{\dot{\tau }}{M}\right)}{\partial \tau }=\frac{\mathrm{kT}}{b}\;∙\;\mathrm{slope}$$

(7) $$\sigma_{a}=\frac{\mathrm{kT}}{b}\;∙\;\sigma_{\mathrm{slope}}$$

Now, when plotting the information in a Haasen plot, we need to compute and plot *b*²/Δ*a*, for which the corresponding error *σ*_b²/Δa_ is given (via the usual law on relative error) as:

(8) $$\sigma_{b^{2}\;/a}=-\frac{b^{2}}{a}∙\frac{\sigma_{\mathrm{slope}}}{\mathrm{slope}}$$

(the sign − being introduced simply to compensate for the negative value of the slope).
